# Prediction of Visual Field Progression in Patients with Primary Open-Angle Glaucoma, Mainly Including Normal Tension Glaucoma

**DOI:** 10.1038/s41598-017-15267-y

**Published:** 2017-11-08

**Authors:** Koji Nitta, Ryotaro Wajima, Gaku Tachibana, Sachie Inoue, Tatsuya Ohigashi, Naomi Otsuka, Hiroaki Kurashima, Kazunori Santo, Masayo Hashimoto, Hidetoshi Shibahara, Mai Hirukawa, Kazuhisa Sugiyama

**Affiliations:** 1 Fukui-ken Saiseikai Hospital, Fukui, Japan; 2 Kanazawa University Graduate School of Medical Science, Kanazawa, Japan; 3 Crecon Medical Assessment Inc., Tokyo, Japan; 4 Santen Pharmaceutical Co., Ltd., Osaka, Japan

## Abstract

An objective method to predict individual visual field progression will contribute to realise personalised medication. The purpose of this study was to establish a predictive formula for glaucomatous visual field progression in patients with Primary open-angle glaucoma, mainly including normal tension glaucoma. This study was a large-scale, longitudinal and retrospective study including 498 eyes of 312 patients visiting from June 2009 to May 2015. In this analysis, 191 eyes of 191 patients meeting all eligible criteria were used. A predictive formula to calculate the rate of glaucomatous visual field progression (mean deviation slope) was obtained through multivariate linear regression analysis by adopting “Angle of Retinal Nerve Fibre Layer Defect” at the baseline, “Vertical Cup-Disc ratio” at the baseline, “Presence or absence of Disc Haemorrhage” during the follow-up period, and “Mean IOP change (%)” during the follow-up period as predictors. Coefficient of determination of the formula was 0.20. The discriminative ability of the formula was evaluated as moderate performance using receiver operating characteristic analysis, and the area under the curve was approximately 0.75 at all cut-off values. Internal validity was confirmed by bootstrapping. The predictive formula established by this type of approach might be useful for personalised medication.

## Introduction

Glaucoma is a primary cause for visual disorders^1–3^, and the only evidence-based treatment is intraocular pressure (IOP)-reducing therapy^4–6^. In the Guidelines for Glaucoma (3^rd^ edition) by the Japan Glaucoma Society, Terminology and Guidelines for Glaucoma (4^th^ edition) by the European Glaucoma Society, and Primary Open-Angle Glaucoma Preferred Practice Pattern Guidelines by the American Academy of Ophthalmology, it is recommended that IOP-reducing therapy should be performed by setting the target IOP for individual patients based on the risk factors, stage of disease, and life expectancy^7–9^. However, no method to predict visual field progression or lifetime prognosis of individual patients has been established, and treatment is conducted based on physicians’ subjective evaluation. Therefore, we need an objective method to predict individual visual field progression or lifetime prognosis for setting an appropriate target IOP. These methods will contribute to consideration of optimal medical decisions, practices and/or interventions based on the risk factors in individual glaucoma patient.

Recently, several formulas to predict the rate of visual field progression through statistical analysis using clinical data were reported. Moraes *et al*. indicated a formula to predict the rate of change in the mean deviation (MD) using the clinical data from the New York Glaucoma Study^10^. Similarly, Ernest *et al*. prepared a formula to predict the rate of change in the visual field index using the clinical data from the Dutch Research Project on Treatment Outcome in Glaucoma Patients Study^11^.

Primary Open-angle glaucoma (POAG) is classified into two types: primary open-angle glaucoma (high tension glaucoma) (POAG (HTG)), with an IOP of >21 mmHg, and normal tension glaucoma (NTG), with an IOP of ≤21 mmHg. POAG (HTG) and NTG are same spectrum as glaucomatous optic neuropathy (GON), but the onset and progression of GON are observed at a lower IOP in NTG patients^12^. Based on the results of previous epidemiological studies, the prevalence of NTG substantially differs among regions, and NTG accounts for the greater portion of POAG patients in Japan/Asia^13,14^. As described above, there have been studies on formulas to predict the rate of visual field progression, but no study involving Japanese/Asian patients with a high prevalence of NTG has been conducted. Predictive formula for these patients will contribute adequate IOP-reducing therapeutic strategies, improving their quality of life.

In this study, we investigated a predictive formula regarding the rate of visual field progression using the clinical data from glaucoma patients, including a high percentage of NTG, and performed internal validation of the formula.

## Results

### Candidate Variables

**Figure 1:**
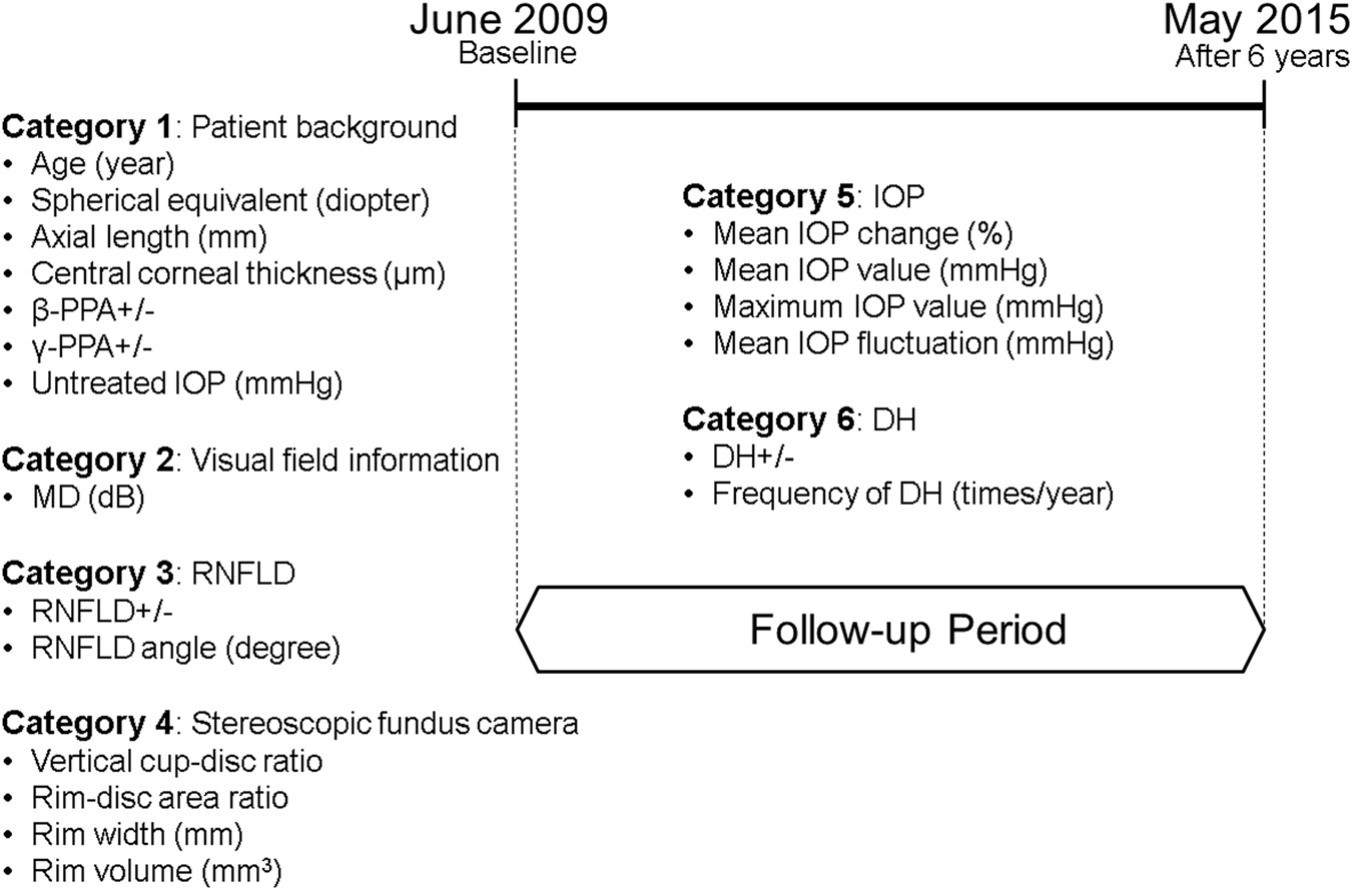
Candidate variables in the analysis. Twenty candidate variables in 6 categories were included in this analysis. Baseline value of variables in category Patient background, Visual field information, RNFLD and Stereo fundus camera were adopted in the analysis. Variables in category IOP and DH were calculated from these values obtained during follow-up period.

The candidate variables used in this study are summarized in Fig. 1. These variables were corrected at baseline or during routine follow-up examinations. Measuring the IOP and optic disc observation were conducted every 2.7 ± 0.8 months. Measuring the visual field with a Humphrey Field Analyzer (HFA) was conducted every 6.7 ± 1.7 months (11.0 ± 3.6 times during follow-up). According to Fleiss, intra-class correlation coefficient (ICC) > 0.75, 0.40 ≤ ICC ≤ 0.75, and ICC < 0.4 are considered to be excellent, moderate, and poor reliability, respectively^15^. The reproducibility of measuring the retinal nerve fibre layer defect (RNFLD) angle by the three raters showed excellent intra-rater (ICC = 0.993, 95% confidence intervals (CI) = 0.962–1.000) and inter-rater (ICC = 0.986, 95% CI = 0.910–0.999) (all P < 0.0001).

### Exploratory Analysis

**Figure 2:**
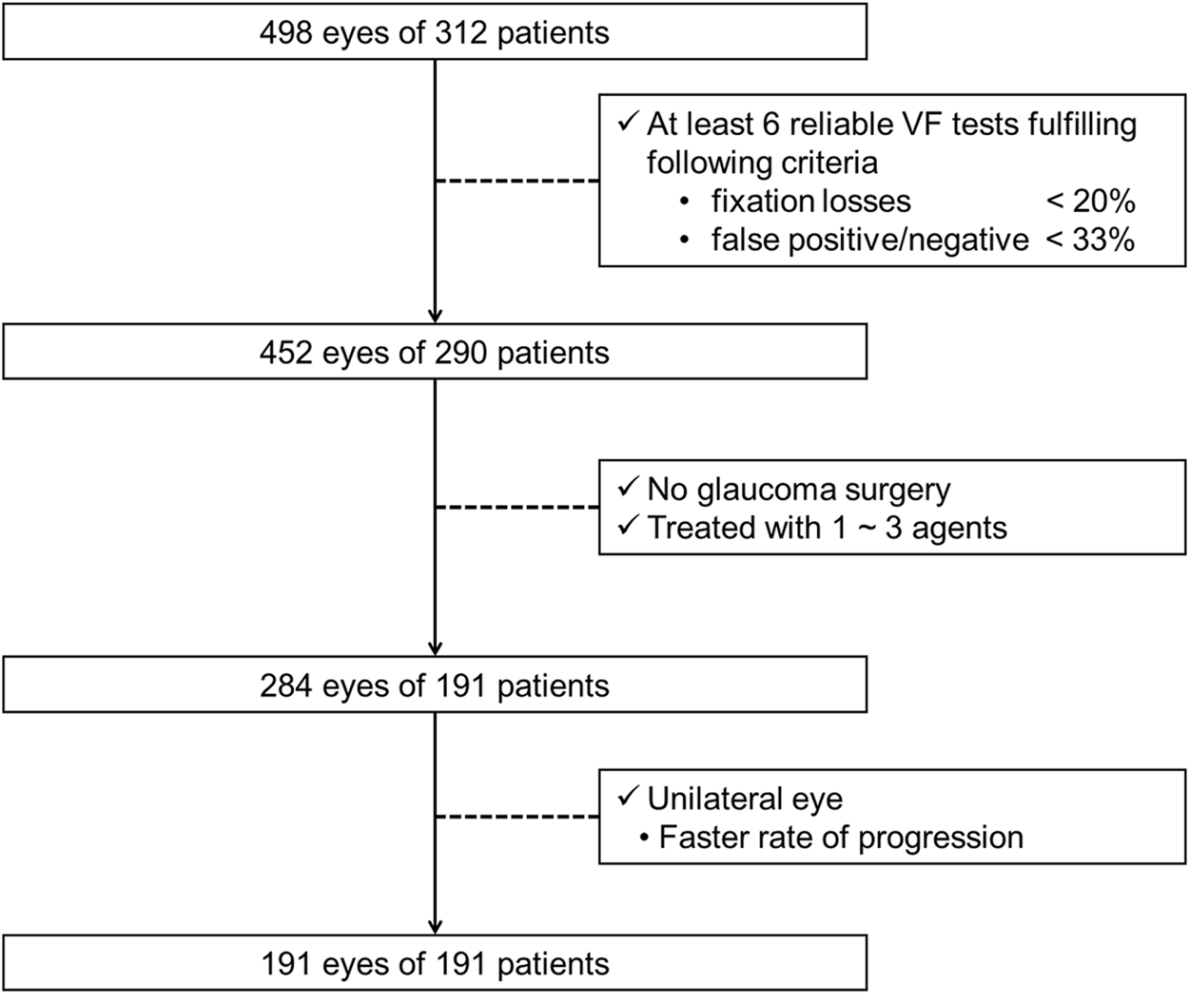
Selection scheme of eyes for the analysis. Study subject were selected through the above 3 steps. 191 eyes of 191 patients were selected from 498 eyes of 312 patients in this scheme.

In this study, 498 eyes of 312 patients were included and 284 eyes of 191 patients met all eligible criteria. A flow chart of the selection process of eyes for the analysis is shown in Fig. 2. The correlation between the MD slope (dB/year) and each variable in only the worse eye (191 eyes of 191 patients) was better than that in worse eye and better eye (284 eyes 191 patients). Therefore the sample of worse eye (191 eyes of 191 patients) was selected as the development sample. The comparison of MD slope between binary variables is summarized in Table 1. The mean MD slope of eyes with RNFLD at baseline (−0.32 dB/year) was significantly lower than that of eyes without RNFLD (−0.18 dB/year) (p < 0.001). The mean MD slope of eye with dis haemorrhage (DH) during the follow-up period (−0.41 dB/year) was significantly lower than that of eyes without DH (−0.26 dB/year) (p < 0.001). The relationships between MD slope and each variable at baseline or during the follow-up period are summarized in Table 2. The correlations between the MD slope and untreated IOP, RNFLD angle, vertical cup-disc ratio (C/D), rim-disc area ratio, rim width, rim volume, and frequency of DH were significant (p < 0.05). The distribution of the MD slope against the key predictive variables (RNFLD angle, rim width, and vertical C/D) are shown in Supplementary Fig. S1

**Table 1:** Comparison of MD slope between each group determined by categorical parameter.

Study eyes (n = 191)		
	MD slope (dB/year)	p-value*
PPA (β/γ/β and γ)	β: −0.32 ± 0.43 γ: −0.37 ± 0.41 β and γ: −0.30 ± 0.44	0.676
RNFLD +/− at baseline	RNFLD(+): −0.32 ± 0.44 RNFLD(−): −0.18 ± 0.42	<0.001
DH +/− during follow-up period	DH(+): −0.41 ± 0.47 DH(−): −0.26 ± 0.42	<0.001

Values are shown as mean ± SD.

MD, Mean deviation; PPA, Peripapillary atrophies; DH, Disc haemorrhage; RNFLD, Retinal nerve fibre layer defect;

*Wilcoxon Signed-rank test or Kruskal-Wallis Test.

**Table 2:** Relationship between MD slope and parameters obtained at baseline/follow-up period.

	Study eyes (n = 191)		
		r	p-value
Baseline	Age (year)	−0.136	0.061
	Untreated IOP (mmHg)	0.192	0.01
	Central corneal thickness (μm)	0.002	0.979
	Axial length (mm)	0.089	0.22
	Spherical equivalent (dioptre)	−0.026	0.723
	MD (dB)	0.113	0.121
	RNFLD angle (degree)	−0.225	0.008
	Vertical cup-disc ratio	−0.300	<0.001
	Rim-disc area ratio	0.246	0.001
	Rim width (mm)	0.308	<0.001
	Rim volume (mm³)	0.177	0.014
Follow-up period	Mean IOP change (%)	−0.125	0.094
	Mean IOP value (mmHg)	0.119	0.101
	Mean IOP fluctuation (mmHg)	0.016	0.831
	Maximum IOP value* (mmHg)	0.109	0.133
	Frequency of DH (times/year)	−0.202	0.005

r, coefficient of correlation; IOP, Intraocular pressure; MD, Mean deviation; RNFLD, Retinal nerve fibre layer defect; DH, Disc haemorrhage;

*Data from IOP in range of the 25th percentile during the follow-up period.

### Study Population for Model Development

**Figure 3:**
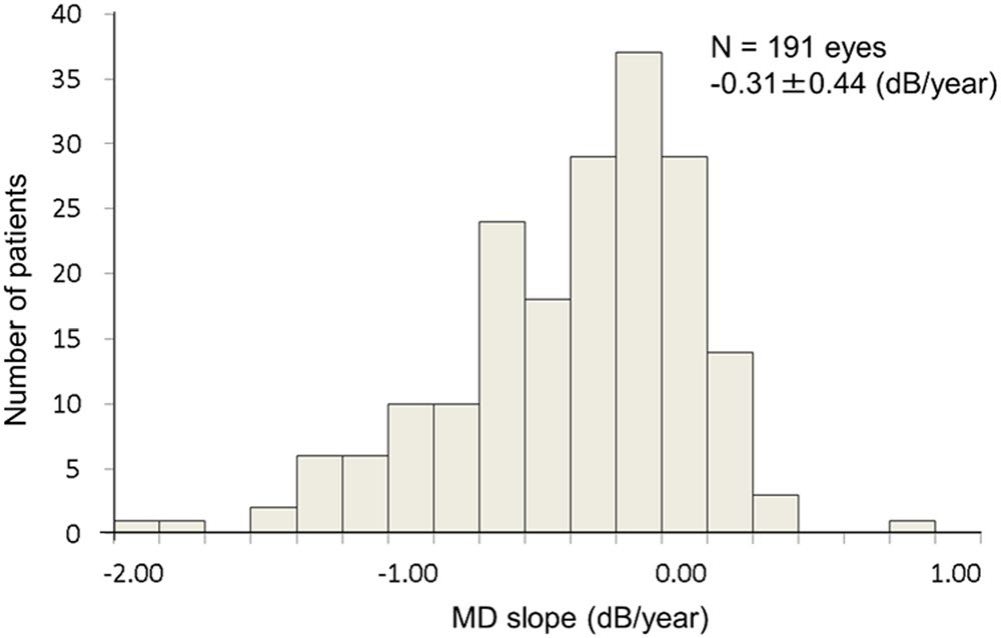
Distribution of individual MD slope during follow-up period. MD slope was calculated assuming linearity by plotting the MD values obtained from reliable visual field tests. Mean MD slope of 191 eyes was −0.31 ± 0.44 (dB/year).

The demographics and ocular characteristics of patients are summarized in Table 3. More detailed information is shown in Supplementary Table S1. A total of 191 eyes of 191 patients, consisting of 23 POAG (HTG) and 168 NTG, with age of 60.85 ± 10.32 years old at baseline, were included in the model development. Over half of the eyes were early stage glaucoma (−6 dB < MD) at baseline. MD was −6.41 ± 5.18 dB at baseline and −8.12 ± 5.75 dB at final observation. IOP change was −19.71 ± 13.34% and number of eyes with DH was 62 (32.5%) during the follow-up period. The distribution of individual MD slope is shown in Fig. 3, and mean MD slope was −0.31 ± 0.44 (dB/year).

**Table 3:** Demographic/ocular characteristics

Study eyes (n = 191)		
Baseline	Age (year)	60.85 ± 10.32
	Sex (male/female)	123/68
	Diagnosis (POAG (HTG)/NTG)	23/168
	Disease stage* (early/moderate/severe)	108/54/29
	Untreated IOP (mmHg)	16.49 ± 3.95
	Central corneal thickness (μm)	536.98 ± 38.49
	Axial length (mm)	25.16 ± 1.76
	Spherical equivalent (dioptre)	−3.58 ± 3.44
	PPA (β/γ/β and γ)	85/37/64
	MD (dB)	−6.41 ± 5.18
	RNFLD angle (degree)	46.53 ± 25.83
	No. of eyes with RNFLD	167 (87.4%)
	Vertical cup-disc ratio	0.79 ± 0.10
	Rim-disc area ratio	0.45 ± 0.13
	Rim width (mm)	0.19 ± 0.08
	Rim volume (mm³)	0.18 ± 0.11
Follow-up period	Mean IOP change (%)	−19.71 ± 13.34
	Mean IOP value (mmHg)	12.80 ± 1.98
	Mean IOP fluctuation (mmHg)	1.63 ± 0.45
	Maximum IOP value^†^ (mmHg)	14.86 ± 2.29
	No. of eyes with DH	62 (32.5%)
	Frequency of DH (times/year)	0.13 ± 0.26

Values are shown as mean ± SD or frequency.

POAG (HTG), Primary open angle glaucoma (high tension glaucoma); NTG, Normal tension glaucoma; IOP, Intraocular pressure; PPA, Peripapillary atrophies; MD, Mean deviation; RNFLD, Retinal nerve fibre layer defect; DH, Disc haemorrhage;

*Defined using Anderson criterion, Early (−6 dB < MD), Moderate (−12 dB ≤ MD ≤ −6 dB), Severe (−12 dB > MD).

^†^Data from IOP in range of the 25th percentile during the follow-up period.

### Model Development

**Figure 4:**
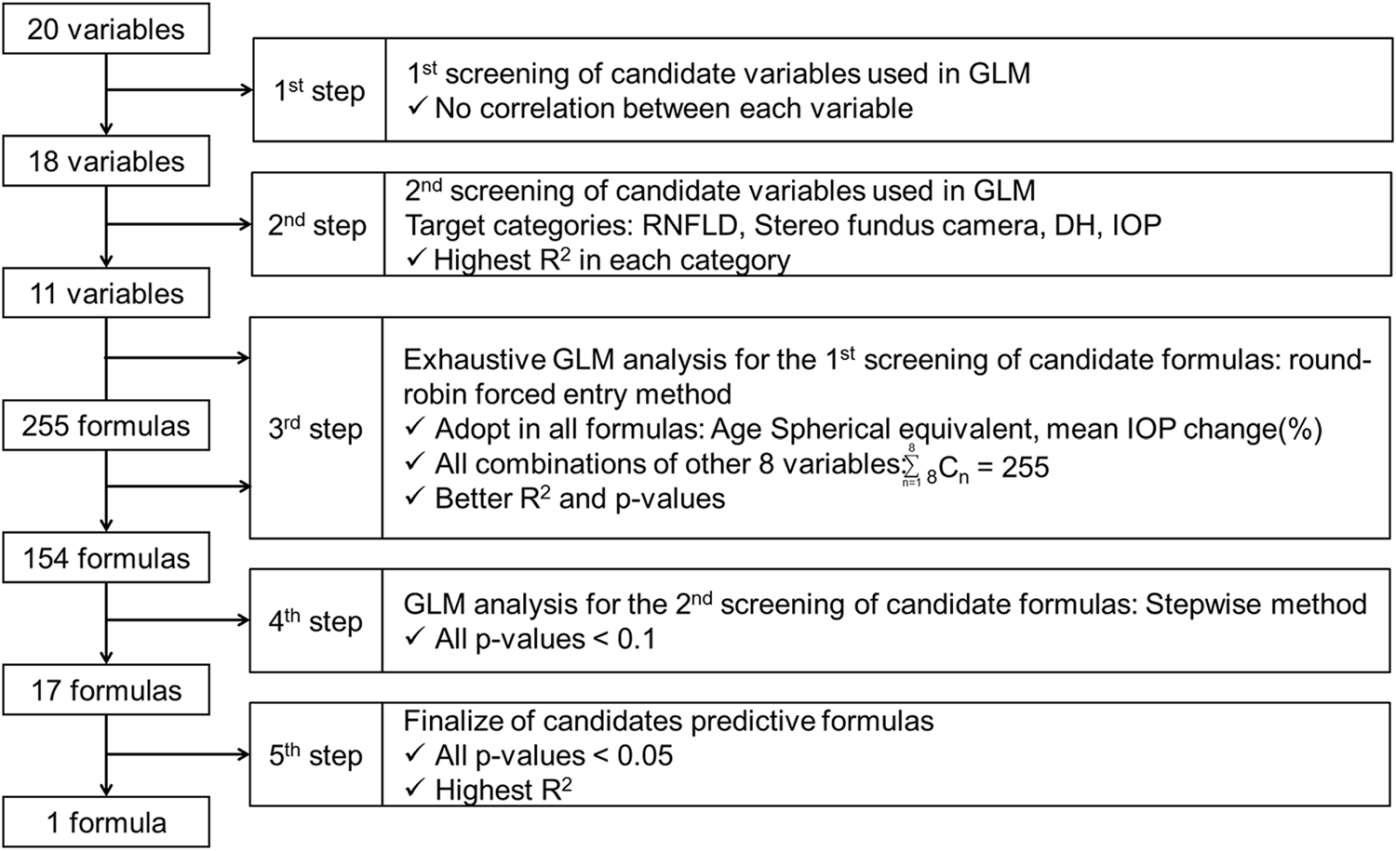
Model development scheme. Model development was performed through the above 5 steps. Candidate variables were refined from 20 to 11 in 1^st^ and 2^nd^ step. Predictive formula was refined from 255 to 1 in 3^rd^, 4^th^ and 5^th^ step.

The prediction model for an individual MD slope was developed through two phases: phase 1; selection of suitable explanatory variables (1^st^ and 2^nd^ step), phase 2; selection of predictive formula (3^rd^, 4^th^, and 5^th^ step). Model development scheme of variables and formulas selection is shown in Fig. 4.

#### 1^st^ step: 1^st^ screening of candidate variables

We conducted screening of candidate variables before conducting multiple regression analysis because the correlation among candidate variables (e.g. vertical C/D, rim-disc area ratio, and rim width) was expected and avoiding multicollinearity was needed. Age, spherical equivalent, untreated IOP, and mean IOP change (%) were regarded as the candidate variables applied to linear regression analysis without any preselection because these are previously reported as risk factors^4–6,16,17^. There was a strong correlation between the untreated IOP and mean IOP change (%) (correlation coefficient (r): −0.707, Pearson’s product-moment correlation coefficient); therefore, the untreated IOP was excluded. In addition, the multicollinearity between remaining 3 variables (age, spherical equivalent, and mean IOP change (%)) and other candidate variables was also evaluated. There was a strong correlation between the spherical equivalent and axial length (r = −0.829, Pearson’s product-moment correlation coefficient); therefore, axial length was excluded from the candidate variables. In this step, 20 variables were refined to 18 variables.

#### 2^nd^ step: 2^nd^ screening of candidate variables

For the refinement of variables, the three variables remained in the 1^st^ step (age, spherical equivalent, and mean IOP change (%)) and each variable in 4 categories (RNFLD, stereo fundus camera, IOP, and DH) were applied in a linear regression analysis using the forced entry method, respectively. The results of analysis demonstrated that the representative variables from the 4 categories were the RNFLD angle at baseline in Category 3, vertical C/D at baseline in Category 4, mean IOP value in Category 5, and presence or absence of DH in Category 6. As the central corneal thickness (CCT), presence or absence of β-peripapillary atrophies (PPA), and presence or absence of γ-PPA in the “patient background” category are different clinical parameters, they were regarded as candidate variables. In this step, 18 variables were refined to following 11 variables: age, spherical equivalent, CCT, presence or absence of β-PPA, presence of absence of γ-PPA, MD at baseline, RNFLD angle, vertical C/D, mean IOP change (%), mean IOP value, and presence of absence of DH.

#### 3^rd^ step: Exhaustive GLM analysis for the 1^st^ screening of candidate formulas

Exhaustive GLM analysis using the 11 candidate variables selected in the 2^nd^ step was performed by the round-robin forced entry method, and conducted the 1^st^ screening for a candidate predictive formula. Adopting age, spherical equivalent and mean IOP change (%) were adopted in all candidate predictive formulas, hence all combinations of other 8 variables (_8_C_1_ + _8_C_2_ + _8_C_3_ + _8_C_4_ + _8_C_5_ + _8_C_6_ + _8_C_7_ + _8_C_8_ = 255) were evaluated. The selection of formulas was conducted comprehensively considering the following criteria; 1) raising the R^2^ statistical value, 2) lowering the number of variables, 3) including the variables prioritised in 1^st^ screening as possible. As for the results, the 255 candidate predictive formulas were refined to 154 formulas. The candidate predictive formula, including all 11 variables using the round-robin forced entry method is shown in Supplementary Table S2.

#### 4^th^ step: GLM analysis for the 2^nd^ screening of candidate formulas

Concerning the 154 candidate predictive formulas refined in the 3^rd^ step, GLM analysis was performed using the stepwise method. Predictive formulas which p-value of all variables was <0.1 were selected. As the results, total 17 candidate predictive formulas were selected.

#### 5^th^ step: Finalize of candidate formulas

Of the 17 candidate predictive formulas selected in the 4^th^ step, we selected predictive formula with a p-value of <0.05 and the highest R^2^ statistic. The characteristic of selected predictive formula is shown in Table 4.

**Table 4:** The characteristic of selected formula

Variables	Non-standardization coefficient		Standardizing Coefficient	p-value	R^2^	adjusted R^2^
	estimate	SE	estimate			
Intercept	0.581	0.274	—	0.036	0.195	0.170
Baseline RNFLD angle	−0.002	0.001	−0.164	0.048		
Baseline vertical C/D	−1.079	0.365	−0.249	0.004		
Presence of DH	−0.184	0.072	−0.209	0.012		
Mean IOP change (%)	−0.006	0.003	−0.178	0.029		

SE, standard error; R^2^, coefficient of determination.

MD slope (dB/year) = 0.581 + [(Baseline RNFLD angle) × −0.002] + [(Baseline vertical C/D) × −1.079] + [(Presence of DH) × −0.184] + [(Mean IOP change (%)) × −0.006]

Samples with all 4 variables of this formula were obtained from 133 eyes of 133 patients (development samples). The optimism-corrected R^2^ was 0.195, and the adjusted R^2^ was 0.170. Using this predictive formula, the MD slope was calculated based on representative vertical C/D and RNFLD angle for the combination of the presence or absence of DH and mean IOP change (from 0 to −30%) (Table 5).

**Table 5:** Example of predicted MD slope (dB/year).

	0% IOP change				−10% IOP change				−20% IOP change				−30% IOP change			
	RNFLD angle (degree)				RNFLD angle (degree)				RNFLD angle (degree)				RNFLD angle (degree)			
**Predicted MD slope without DH**																
v C/D	30	60	90	120	30	60	90	120	30	60	90	120	30	60	90	120
0.7	−0.23	−0.29	−0.35	−0.41	−0.17	−0.23	−0.29	−0.35	−0.11	−0.17	−0.23	−0.29	−0.05	−0.11	−0.17	−0.23
0.8	−0.34	−0.40	−0.46	−0.52	−0.28	−0.34	−0.40	−0.46	−0.22	−0.28	−0.34	−0.40	−0.16	−0.22	−0.28	−0.34
0.9	−0.45	−0.51	−0.57	−0.63	−0.39	−0.45	−0.51	−0.57	−0.33	−0.39	−0.45	−0.51	−0.27	−0.33	−0.39	−0.45
**Predicted MD slope with DH**																
v C/D	30	60	90	120	30	60	90	120	30	60	90	120	30	60	90	120
0.7	−0.42	−0.48	−0.54	−0.60	−0.36	−0.42	−0.48	−0.54	−0.30	−0.36	−0.42	−0.48	−0.24	−0.30	−0.36	−0.42
0.8	−0.53	−0.59	−0.65	−0.71	−0.47	−0.53	−0.59	−0.65	−0.41	−0.47	−0.53	−0.59	−0.35	−0.41	−0.47	−0.53
0.9	−0.63	−0.69	−0.75	−0.81	−0.57	−0.63	−0.69	−0.75	−0.51	−0.57	−0.63	−0.69	−0.45	−0.51	−0.57	−0.63

MD, Mean deviation; DH, Disc haemorrhage; IOP, Intraocular pressure; RNFLD, Retinal nerve fibre layer defect; v C/D, Vertical cup-disc ratio.

### Validation to Predictive Formula

**Figure 5:**
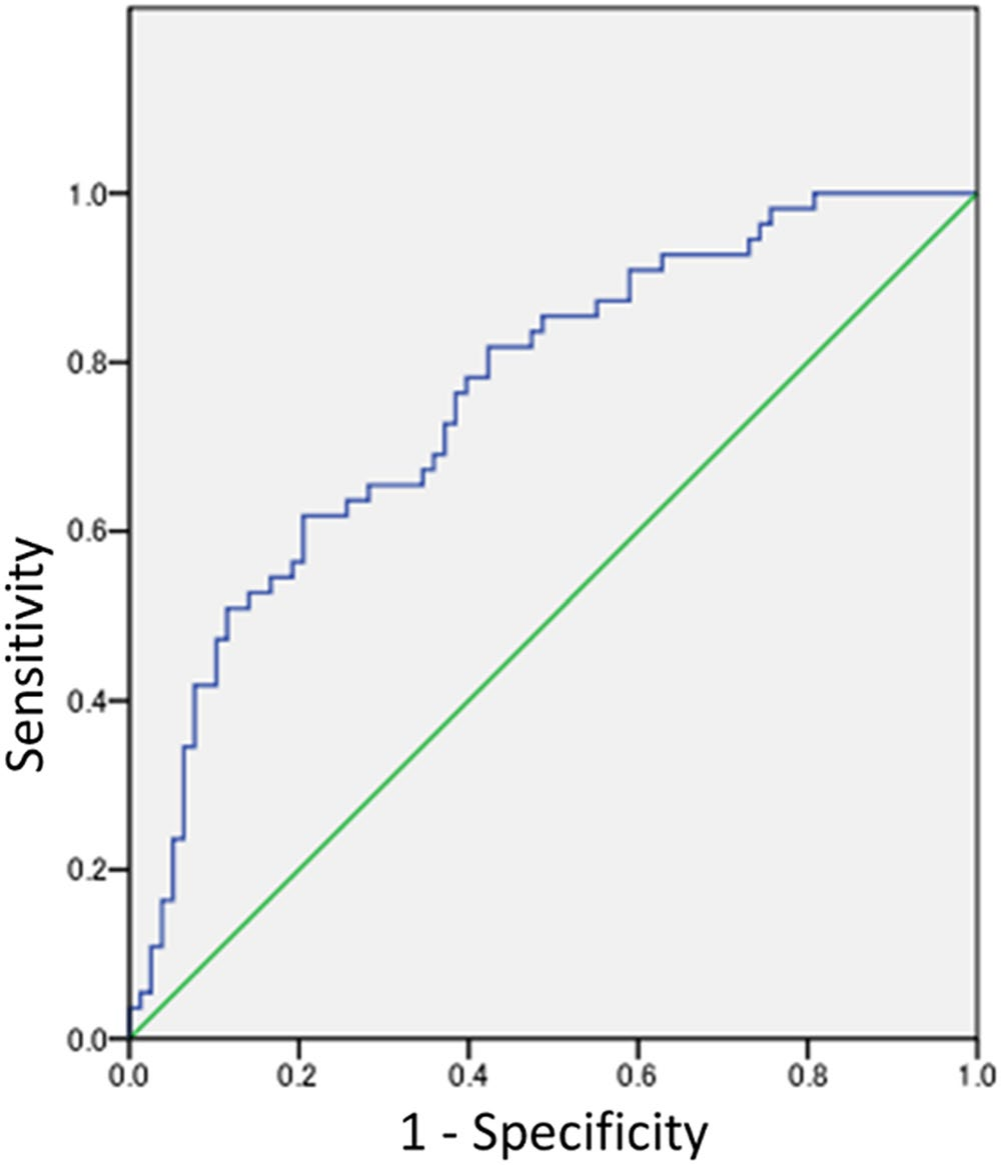
Receiver-operating characteristic curve for detecting the MD slope ≤−0.1 dB/yearn. The AUC for detecting the MD slope ≤−0.1 (dB/year) was 0.762 (95% Confidence Interval: 0.680–0.843).

The discriminative ability was tested by calculating the areas under the curve (AUC) of receiver-operating characteristic (ROC) curves for detecting the MD slope from −0.1 to −0.5 (dB/year). The AUCs for detecting the MD slope were calculated using development sample and bootstrap sample (Table 6). All AUCs at a cut-off value (−0.1 to −0.5 dB/year) exceeded 0.7. The ROC curve to detect a slope of MD ≤ −0.1 (dB/year) is shown in Fig. 5. Using 200 bootstrap samples, the optimism-corrected R^2^ was 0.224 ± 0.051 (adjusted R^2^: 0.200 ± 0.053) and the AUC was calculated ranged from 0.736 to 0.772 for detecting the MD slope from −0.1 to −0.5 (dB/year) (Tables 6 and 7).

**Table 6:** Receiver-operating characteristic curve for detecting the slope using development samples (n = 133) and bootstrap samples (n = 200).

Development samples (n = 133)						Bootstrap samples (n = 200)			
Detected MD slope	Observed Cases	Prevalence (%)	AUC	95% CI		Detected MD slope	Mean Prevalence (%)	AUC	
				Lower limit	Upper limit			Mean	SD
≤−0.1	78	58.60%	0.762	0.680	0.843	≤−0.1	58.60%	0.768	0.039
≤−0.2	71	53.40%	0.763	0.683	0.844	≤−0.2	53.40%	0.769	0.038
≤−0.3	59	44.40%	0.733	0.649	0.818	≤−0.3	44.20%	0.739	0.041
≤−0.4	49	36.80%	0.766	0.684	0.848	≤−0.4	36.80%	0.772	0.043
≤−0.5	45	33.80%	0.73	0.642	0.818	≤−0.5	33.70%	0.736	0.045

MD, Mean deviation; AUC, Area under the curve; SD, standard deviation; CI, Confidence Interval.

**Table 7:** Results of GLM using development samples (n = 133) and bootstrap samples (n = 200).

Development samples (n = 133)		Bootstrap samples (n = 200)			
R²	adjusted R²	R²		adjusted R²	
		Mean	SD	Mean	SD
0.195	0.170	0.224	0.051	0.200	0.053

GLM, generalized linear model; R^2^, coefficient of determination; SD, standard deviation.

## Discussion

In this study, we established a formula to predict the rate of visual field progression in patients with POAG, mainly including NTG using clinical data. We performed single correlation analysis using the MD slope as an objective variable. There were correlations between glaucomatous structural abnormalities and the MD slope. Furthermore, a predictive formula to calculate the MD slope was obtained through multivariate linear regression analysis by adopting the RNFLD angle at baseline, vertical C/D at baseline, presence or absence of DH during the follow-up period, and mean IOP change during the follow-up period as predictors. The accuracy of this formula was confirmed as moderate performance by ROC analysis and internal validation.

Glaucoma is a neurodegenerative disease, and visual field loss associated with glaucomatous structural abnormality is common among POAG (HTG) and NTG. Most of the variables in this study represent these common glaucomatous structural abnormalities, not true disease causes. Therefore, both POAG (HTG) and NTG were included in the development sample. In epidemiological studies in Japan/Asia, it was reported that NTG accounted for 92%^13^ or 77%^18^ of all patients with POAG. Of the 191 patients analysed in this study, NTG accounted for 88%. This percentage was similar to that in the epidemiological studies. Hence, this predictive formula is considered to be appropriate to predict visual field progression in POAG patients in Japan/Asia.

The results of this study suggest that glaucomatous structural abnormalities, vertical C/D and RNFLD angle, DH are risk factors accelerating the rate of visual field progression. Sehi *et al*. investigated the relationship between glaucomatous structural abnormalities at baseline and the visual field progression involving the patients obtained in the Advanced Imaging for Glaucoma Study, suggesting that structural abnormalities of the optic nerve head and presence of RNFLD accelerate the progression^19^, as indicated in our study. In our previous study, the enlargement of the RNFLD angle and rate of visual field progression were accelerated in NTG patients with DH using long-term follow-up data^20^. As shown in Table 5, when utilizing the predictive formula obtained in this study, the rate of visual field progression is comprehensively calculated based on the RNFLD angle at baseline, vertical C/D at baseline, and presence or absence of DH during the follow-up period. Thus, it must be important to carefully examine glaucomatous structural abnormalities or the presence or absence of DH using stereoscopic fundus photographs and fundus imaging devices to evaluate the risk of visual field progression in individual patients.

A large-scale, randomized, comparative study demonstrated that IOP-reducing therapy was the only evidence-based treatment method for not only POAG (HTG) but also NTG patients^4–6,21^. In the Collaborative Normal-Tension Glaucoma Study, the visual field progression was compared between a treatment group (IOP reduction ≥ 30%) and an untreated group, and the effectiveness of IOP-reducing therapy for NTG was confirmed^21^. Similarly, we clarified the influence of IOP-reducing therapy on the rate of visual field progression in NTG patients through this study. Concretely, the regression coefficient of the mean IOP change was estimated to be −0.006; therefore, in case the target IOP change is set as −30%, inhibitory effect on rate of visual field progression is calculated as −0.006 × (−30) = 0.18 (dB/year). The influence of 30% IOP-reducing therapy on the rate of visual field progression is shown in Table 5, which suggests the effectiveness of IOP-reducing therapy. As principal, in case the target IOP is not sufficiently achieved, or the visual field progression is observed, additional IOP-reducing therapy should be conducted. However, potent IOP-reducing therapy should be considered earlier for high-risk patients.

In this study, we conducted internal validation using original and bootstrap sample which is classified as a resampling method among statistical estimation procedures^22^. As a result, both examinations demonstrated that the R^2^ for the predictive formula obtained was approximately 0.2. Furthermore, we performed ROC analysis using these samples, establishing the cut-off value of the MD slope as −0.1 to −0.5 (dB/year). At all cut-off values, the AUC was approximately 0.75, confirming that the accuracy is moderate: AUC: 0.5 to 0.7, less accurate; 0.7 to 0.9, moderately accurate; and 0.9 to 1.0, highly accurate^23,24^. The R^2^ for the predictive formula obtained in this study (0.2) was higher than previous reports (Moraes *et al*. R^2^ = 0.14, Ernest *et al*. R^2^ = 0.10), and our AUC (0.75) was similar to precious reports (Moraes *et al*. AUC = 0.78, Ernest *et al*. AUC = 0.76)^10,11^.

These are some limitations in this study. First, as the primary purpose of this study was to find a formula with optimal accuracy, we examined the development sample and combination of several variables, and selected an optimal predictive formula. Although only worse eyes were included in the development sample, this predictive formula was considered to be reasonable because predicting visual filed progression of worse eye is meaningful for clinical use. Variables for the rate of visual field progression that negatively affects the accuracy of the predictive formula were not selected. Therefore, the involvement of risk factors that are not contained in this predictive formula in the rate of visual field progression is not disclaimed. In addition, glaucoma is a multifactorial disease, and presence of uncertain risk factors in the progression of the condition is suggested^12^. The predictive formula obtained in this study might not completely explain the rate of visual field progression. Second, in the step of variable selection for model arrangement, there was a strong correlation between variables representing refraction, that is, the axial length and spherical equivalent (r = −0.829). To avoid multicollinearity, we should select either one. The intraocular lens influences the spherical equivalent; therefore, the axial length may more accurately reflect the condition. However, in this study, the spherical equivalent in pseudophakic eyes was measured in only 7.3% of the patients; therefore, to establish a commonly available predictive formula, the spherical equivalent was selected as a candidate variable in the 1^st^ step. Third, the stepwise selection of candidate variables is prone to cause overfitting of the model to the study population and might limit its generalizability. Fourth, DH is easily missed on fundus examination if physicians are not specially examining for it. The prevalence of DH was reported as 20.5% or 33.3% in Japanese/Asian NTG patients^25,26^. In our daily practice, optic disc was carefully observed using stereo fundus camera and DH was observed in 32.5% of the development sample. Kitazawa *et al*. reported that 92% of DHs exist at least for 4 weeks and over half of them were recurrent^25^. It could be considered that not all DHs were detected in this study because the fundus examination was performed mostly every three months. Fifth, the data obtained from stereo fundus camera were used as baseline parameter. Stereo fundus camera has installed in our hospital at 2010; therefore we considered the value from earliest examination during follow-up period as a baseline value in this analysis. Sixth, corresponding evaluation between structural abnormality and functional disorder is important in daily practice and thus indices based on this concept were reported^27,28^. In this study, we also performed the preliminary analysis of structural-functional relationships using the hemifield total deviation slope, but the results were not statistically superior to those of the MD slope. Thus, we selected the MD slope as an objective variable because it is more commonly used in glaucoma management. Seventh, concerning the confirmation of the accuracy, internal validation using the data obtained in this study alone was conducted, because we had no eligible data for conducting external validation. Therefore, in the future, the accuracy of this predictive formula should be confirmed by performing external validation using data obtained in other institutions or a prospective study.

In conclusion, a formula to predict the rate of visual field progression in patients with POAG, mainly including NTG was obtained using clinical data. We consider this formula could be moderately predictive and useful for establishing an appropriate target IOP for individual patients and selecting therapeutic strategies, thereby contributing to adequate IOP-reducing therapy.

## Materials and Methods

### Study Population

This study was a large-scale, longitudinal, retrospective study. The data was obtained from POAG (HTG) and NTG patients visiting Fukui-ken Saiseikai Hospital regularly from June 2009 to May 2015. Patients received routine follow-up examinations mostly every three months and standard medical care prescribed by only one ophthalmologist (N.K.). This study was approved by the institutional review board. And this study was conformed to the tenets of the Declaration of Helsinki and the Ethical Guidelines for Medical and Health Research Involving Human Subjects (issued on December 12, 2014 by the Ministry of Education, Culture, Sports, Science and Technology and the Ministry of Health, Labour and Welfare established the Ethical Guidelines for Epidemiological Research of Japan). The institutional review board also approved the use of “opt-out” patient consent before inclusion in the study.

### Candidate Variables

**Figure 6:**
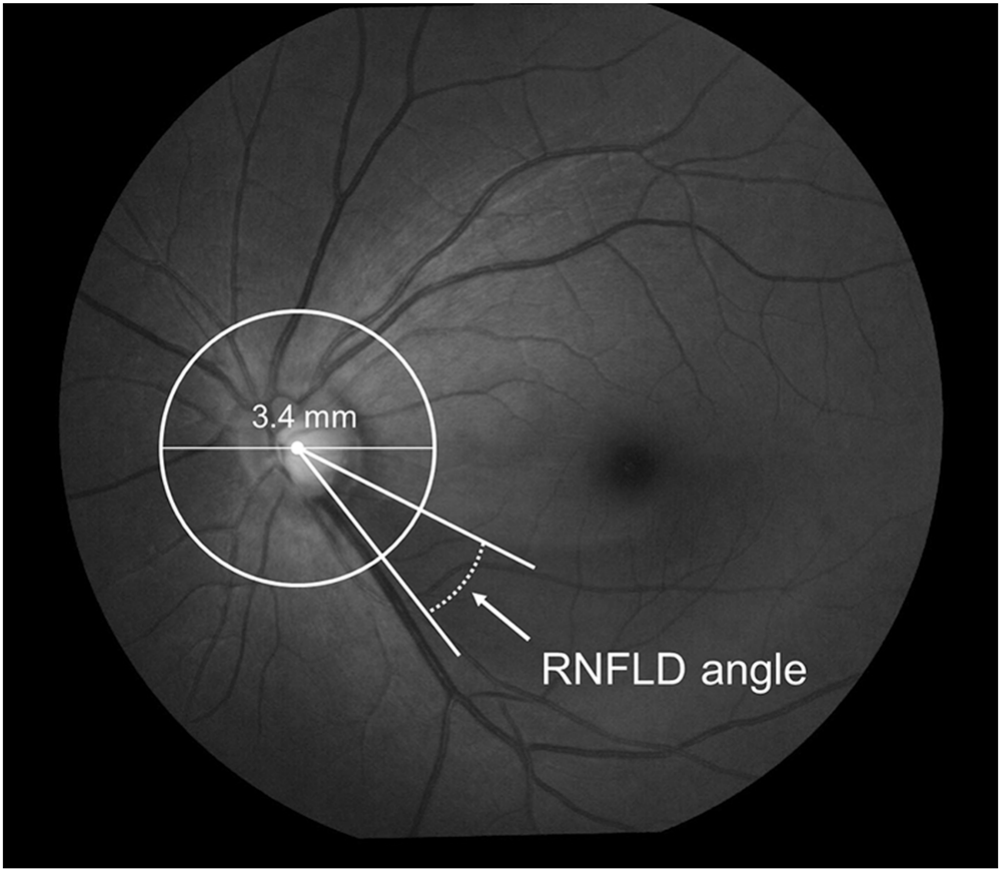
Definition of RNFLD Angle. Draw a circle with a diameter of 3.4 mm centred at the disc centre. Determine the points where the circle intersects the RNFLD. Draw a line between the intersecting point and the disc centre. RNFLD angle is defined as the angle between the two resulting lines.

At each visit, patients had ocular examinations, including visual acuity, IOP measurements with a Goldmann applanation tonometer, and evaluations by slit-lamp, gonioscope, indirect ophthalmoscope, and stereo fundus camera. All visual fields in this study were measured with SITA-standard 24-2 or 30-2 tests using the HFA (Carl Zeiss Meditec, USA). The visual field was measured mostly every six months. The PPA was evaluated based on the status of Bruch’s membrane from 3 B-scan images obtained from optical coherence tomography^29^, RS-3000 advance (Nidek, Co., Ltd. Japan). We determined the angle of the RNFLD (Fig. 6). The subject’s identity and the other test results were masked to the examiner. RNFLD was observed using a fundus digital camera (VX-10, Kowa Optimed, Inc. Japan) and coloured fundus photographs with only the blue ingredient extracted obtained from the Nidek Advanced Vision Information System (Nidek, Co., Ltd. Japan) were used in this study. In case RNFLD existed superior and inferior, we summed both RNFLD angles. Measurements of RNFLD angle were performed three times for each photograph and the mean value of the measurements was used in the analysis. The intra-rater and inter-rater reproducibility were evaluated in terms of the ICC and 95% CI’s by measurement of the RNFLD angle using same photograph by same or different raters. The optic disc was observed through a +14 dioptre lens with the pupil was not dilated, and confirmed whether DH existed or not at each visit. In case the presence of DH was suspected, the fundus was observed again with the pupil dilated, then photographed using a fundus digital camera (VX-10, Kowa Optimed, Inc. Japan) or stereo fundus camera (Nonmyd WX, Kowa Optimed, Inc. Japan). Optic nerve head parameters such as vertical C/D and rim volume were measured using the 3D analysis system (VK-2 WX, Kowa Optimed, Inc. Japan). After the manual determination of disc and cup edge in the 3D image, these parameters are automatically measured by this software.

Eligible patients fulfilled all the following criteria for at least one eye: 1) 20 years or older at the start of the follow-up period (baseline), 2) observed mostly every three months during the follow-up period, 3) diagnosed as POAG (HTG) or NTG at baseline, 4) accurately measurable IOP (excluding those with history of keratorefractive surgery, ocular injury, and keratoconus, etc.), 5) observable fundus without mydriatic, 6) no history or complication of retinal disease (however, if the investigator considered that there was no influence on the assessment of visual field progression during the survey period, the patient was enrolled), and 7) had at least 6 reliable visual field tests during follow-up period. POAG (HTG) and NTG were diagnosed according to the guidelines of the Japan Glaucoma Society and the European Glaucoma Society^7,30^. The diagnostic criteria for POAG (HTG) were 1) more than 21 mmHg IOP mostly three times without any glaucoma medication. 2) open-angle, 3) GON with corresponding visual field loss, and 4) the absence of other optic neuropathies. The diagnostic criteria for NTG were 1) 21 mmHg or less IOP mostly three times without any glaucoma medication. 2) open-angle, 3) GON with corresponding visual field loss, and 4) the absence of other optic neuropathies. GON was evaluated according to the guidelines of the Japan Glaucoma Society: 1) the vertical C/D of the optic nerve head is 0.7 or more, 2) the rim width at the superior portion (11-1 hours) or inferior portion (5–7 hours) is 0.1 or less of the disc diameter, or 3) the difference of the vertical C/D is 0.2 or more between both eyes, or 4) a RNFLD is found^7^.

As a parameter of visual field loss progression, the MD slope (dB/year) per eye was calculated using data on reliable visual field tests at least ≥ 6 points meeting the following criteria: 1) fixation losses < 20%, 2) false positive or false negative < 33%. MD slope was calculated assuming linearity by plotting the MD values obtained from reliable visual field tests. Patients having a history of glaucoma surgery, those who did not receive any treatment during the follow-up period, or treatment with over 4 agents were excluded.

The IOP change (%) was calculated as [(IOP value of each measurement point) − (baseline IOP values)]/(baseline IOP value) × 100. The mean IOP change (%) was calculated as (sum of the IOP change (%) at each measurement point during the follow-up period)/(total number of measurement). The mean IOP value and its fluctuations were defined as the mean and SD of IOP values during the follow-up period. The maximum IOP value was defined as the mean of upstream 25% values of IOP during the follow-up period. The frequency of DH was defined as (total number of DH during the follow-up period)/(follow-up period). In case DH was observed more than one position in an examination, the number of appearance was summed.

The rate of visual field progression was expressed by the MD slope for each eye. The following 20 variables in 6 categories were included as candidate variables for the MD slope in the evaluation of the prediction model: Category 1) Patient background: age at baseline (year), spherical equivalent (dioptre), axial length (mm), CCT (μm), presence or absence of β-PPA, presence or absence of γ-PPA, and untreated IOP (mmHg), Category 2) Visual field information: MD at baseline (dB), Category 3) RNFLD: presence or absence of RNFLD and its angle (degree), Category 4) Stereo fundus camera: vertical C/D, mean rim width of the upper and lower sides (mm), rim-disc area ratio, and rim volume (mm^3^), Category 5) IOP: mean IOP change (%), mean IOP value (mmHg), maximum IOP value (mmHg), and mean IOP fluctuation (mmHg), Category 6) DH: presence or absence of DH, and its frequency of during follow-up period (times/year). Only the baseline value was used in Category 1 to 4 because the object of this study was to establish the predictive model estimates of the MD slope in the early phase of treatment. Meanwhile, the follow-up value was used in Category 5 and 6 because to evaluate the effect of IOP-lowering treatment was one of the aims of this study and the presence of DH could not be estimate in a single observation.

### Exploratory Analysis

To evaluate the relationship between individual MD slope and each variable, univariate analysis was conducted. Numerical or continuous variables, such as mean IOP change, RNFLD angle, or frequency of DH, were determined by the correlation with MD slope using Pearson’s product-moment correlation coefficient or Spearman’s rank-correlation coefficient. The relationship between the MD slope and each binary variable, including the sex and presence or absence of DH, was determined using Wilcoxon’s signed-rank test, and the nominal variables of 3 values or more, presence or absence of β-PPA and/or γ-PPA, was analysed using the Kruskal-Wallis test. The p-value of <0.05 was considered as significant. The variables for model development were selected through result of univariate analysis.

### Model Development

Development of prediction model for an individual MD slope was conducted in two phases: phase 1; selection of suitable explanatory variables, phase 2; selection of predictive formula. The objective of this study was to construct a predictive formula based on systematic screening of candidate variables. Thus, relationships between individual MD slope and each 20 variable collected in phase 1 and correlations among those variables were assessed.

In phase 2, predictive formula was constructed from exhaustive combinations of variables narrowed in phase 1, and most reasonable predictive formula from the aspects of statistics and practical applicability was selected.

A multivariate linear regression analysis (generalized linear model, GLM) was applied to develop a model to predict an individual MD slope with candidate variables. Multivariate analysis expresses one objective variable with two or more explanatory variables. Model development was performed following 5 steps. The model development was performed using IBM SPSS Statistics 18 software.

#### 1^st^ step: 1^st^ screening of candidate variables

Age, spherical equivalent, untreated IOP, and mean IOP change (%) were regarded as the candidate variables applied to linear regression analysis because these are known as risk factors^4–6,16,17^. In the case that the high correlation between the explanatory variables, i.e. multicollinearity, is observed, there is a possibility that prediction accuracy would be reduced. Therefore, in this step, the multicollinearity among the above 4 candidate variables was evaluated.

#### 2^nd^ step: 2^nd^ screening of candidate variables

For the refinement of variables, the variables adopted in the 1^st^ step and each variable in 4 categories (RNFLD, stereo fundus camera, IOP, DH) involving several candidate variables were applied in a linear regression analysis using the forced entry method, respectively. A variable with a highest coefficient of determination (R^2^) was selected as a representative variable of each category.

#### 3^rd^ step: Exhaustive GLM analysis for the 1^st^ screening of candidate formulas

Exhaustive GLM analysis using the variables preselected in the 1^st^ step and 2^nd^ step was performed by the round-robin forced entry method for 1^st^ screening of a candidate predictive formula. The forced entry method is a method of forcibly incorporating all variables as explanatory variables to construct a multiple regression formula.

#### 4^th^ step: GLM analysis for the 2^nd^ screening of candidate formulas

GLM analysis of the candidate predictive formula refined in the 3^rd^ step was performed using the stepwise method, for the 2^nd^ screening of the formula. The stepwise method is a method of constructing an optimal multiple regression formula by adding or removing one explanatory variable step by step.

#### 5^th^ step: Finalization of candidate formulas

Final predictive formula was selected referring p-value of variables and R^2^ of the formula.

### Validation of Predictive Formula

The discriminative ability was evaluated using a development sample of the final model. The discriminative ability was tested by calculating the AUC of ROC curves. A value of AUC 0.5 indicates that the model has no discriminative ability to identify progressive patients and 1.0 indicates that has perfect discriminative ability^31^. Furthermore, the model was internally validated using 200 bootstrap samples from 191 eyes by calculating the expected R^2^ and AUC. Bootstrap is one of random sampling method with replacement. The size of bootstrap sample is same as original sample, but the composition element is different. The validation was performed using IBM SPSS Statistics 18 software.

### Data Availability

The dataset generated during the current study is available from the corresponding author on reasonable request.

## Electronic supplementary material

Supplementary Information
