# Supplementary material for: Prediction of Visual Field Progression in Patients with Primary Open-Angle Glaucoma, Mainly Including Normal Tension Glaucoma
Source: Sci Rep. 2017 Nov 8;7:15048. doi: 10.1038/s41598-017-15267-y (PMC5678075; doi:10.1038/s41598-017-15267-y)

[Title]

Prediction of Visual Field Progression in Patients with Primary Open-Angle Glaucoma, Mainly Including Normal Tension Glaucoma

[Authors]

Koji Nitta,[1,2,*,+] Ryotaro Wajima,[1,2,+] Gaku Tachibana,[1,2] Sachie Inoue,[3] Tatsuya Ohigashi,[4] Naomi Otsuka,[4] Hiroaki Kurashima,[4] Kazunori Santo,[4] Masayo Hashimoto,[4] Hidetoshi Shibahara,[3] Mai Hirukawa,[3] Kazuhisa Sugiyama[2]

1: Fukui-ken Saiseikai Hospital, Fukui, Japan

2: Kanazawa University Graduate School of Medical Science, Kanazawa, Japan

3: Crecon Medical Assessment Inc., Tokyo, Japan

4: Santen Pharmaceutical Co., Ltd., Osaka, Japan

*nitta.koji7001@fukui.saiseikai.or.jp

[+]these authors contributed equally to this work

[TABLES]

Table S1. Demographic/ocular characteristics

		Study eyes	
		POAG (HTG) (n = 23)	NTG (n = 168)
Baseline	Age (year)	59.22 ± 10.32	61.07 ± 10.33
	Sex (male/female)	18/5	105/63
	Disease stage* (early/moderate/severe)	16/2/5	92/52/24
	Untreated IOP (mmHg)	24.56 ± 3.87	15.42 ± 2.44
	Central corneal thickness (μm)	544.78 ± 46.99	535.92 ± 37.22
	Axial length (mm)	25.09 ± 1.43	25.17 ± 1.80
	Spherical equivalent (dioptre)	-3.48 ± 3.15	-3.59 ± 3.48
	PPA (β/γ/β and γ)	9/3/10	76/34/54
	MD (dB)	-5.69 ± 5.86	-6.51 ± 5.09
	RNFLD angle (degree)	33.10 ± 19.71	47.80 ± 26.05
	No. of eyes with RNFLD	21 (91.3%)	146 (86.9%)
	Vertical cup-disc ratio	0.79 ± 0.10	0.79 ± 0.10
	Rim-disc area ratio	0.43 ± 0.13	0.45 ± 0.13
	Rim width (mm)	0.20 ± 0.10	0.19 ± 0.08
	Rim volume (mm^3)	0.16 ± 0.11	0.19 ± 0.11
Follow-up period	Mean IOP change (%)	-36.31 ± 11.95	-17.51 ± 11.92
	Mean IOP value (mmHg)	15.03 ± 2.17	12.49 ± 1.75
	Mean IOP fluctuation (mmHg)	2.01 ± 0.57	1.57 ± 0.40
	Maximum IOP value† (mmHg)	17.64 ± 2.66	14.48 ± 1.95
	No. of eyes with DH	8 (34.8%)	54 (32.1%)
	Frequency of DH (times/year)	0.08 ± 0.15	0.14 ± 0.27

Values are shown as mean ± SD or frequency.
POAG (HTG), Primary open angle glaucoma (high tension glaucoma); NTG, Normal tension glaucoma; IOP, Intraocular pressure; PPA, Peripapillary atrophies; MD, Mean deviation; RNFLD, Retinal nerve fibre layer defect; DH, Disc haemorrhage;
* : Defined using Anderson criterion, Early (-6 dB < MD), Moderate (-12 dB ≤ MD ≤ -6 dB), Severe (-12 dB > MD)
† : Data from IOP in range of the 25th percentile during the follow-up period

Table S2. The characteristic of candidate predictive formula including all 11 variables

Variables	B	SE	p-value	R^2	adjusted R^2
Intercept	0.617	0.686	0.370	0.238	0.168
Age	-0.002	0.004	0.667		
Spherical equivalent	-0.018	0.015	0.225		
CCT	-0.001	0.001	0.456		
Presence of β-PPA	-0.095	0.104	0.360		
Presence of γ-PPA	0.044	0.097	0.653		
MD at baseline	-0.002	0.008	0.806		
Baseline RNFLD angle	-0.003	0.001	0.056		
Baseline vertical C/D	-1.026	0.400	0.011		
Mean IOP change (%)	-0.006	0.003	0.036		
Mean IOP value	0.029	0.018	0.115		
Presence of DH	-0.174	0.074	0.021		

SE, standard error; R^2, coefficient of determination

[FIGURE LEGENTDS]

Figure S1. Distribution of MD slope against key predictive parameters

In a graph representing RNFLD angle (a), Vertical cup-disc ratio (b), and rim width (c) on the vertical axis and MD slope on the horizontal axis, the measured values were plotted to create a scatter diagram.

[FIGURES]

Figure S1. Distribution of MD slope against key predictive parameters

a

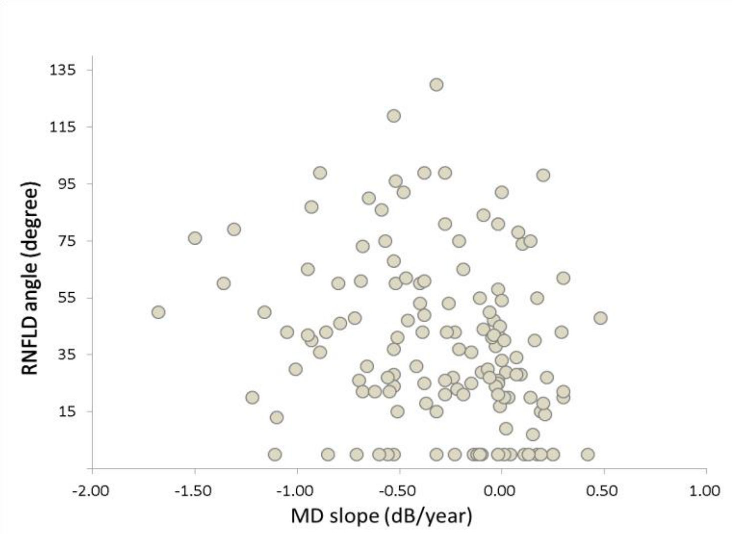

b

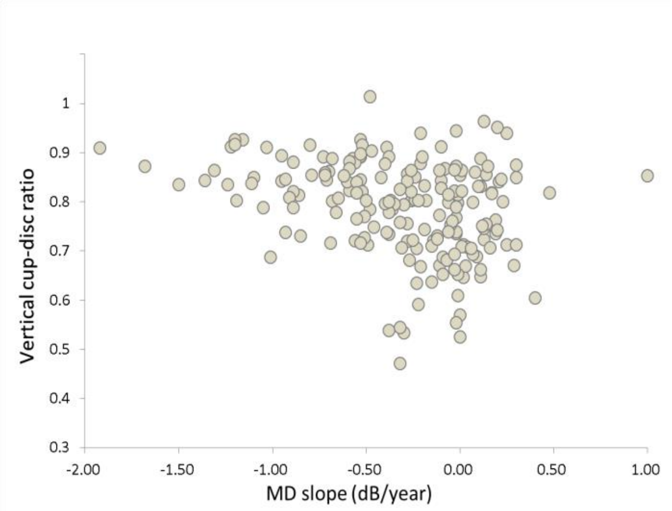

c

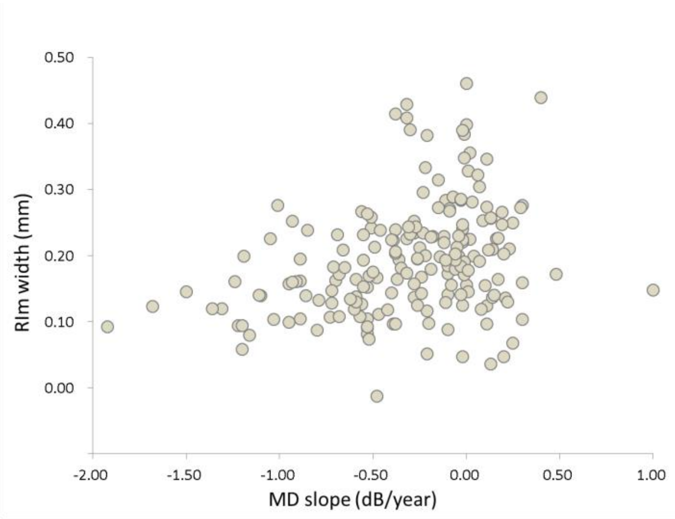

Supplement: Supplementary file 1 — Supplementary Information [file 41598_2017_15267_MOESM1_ESM.pdf]
